# Nonparametric relevance-shifted multiple testing procedures for the analysis of high-dimensional multivariate data with small sample sizes

**DOI:** 10.1186/1471-2105-9-54

**Published:** 2008-01-27

**Authors:** Cornelia Frömke, Ludwig A Hothorn, Siegfried Kropf

**Affiliations:** 1 Department of Biometry, Hannover Medical School, Carl-Neuberg-Str. 1, D-30625 Hannover, Germany; 2 Institute of Biostatistics, Leibniz University of Hannover, Herrenhäuserstr. 2 D-30419 Hannover Germany; 3 Institute for Biometry and Medical Informatics, Otto von Guericke University Magdeburg, Leipziger Str. 44, D-39120 Magdeburg, Germany

## Abstract

**Background:**

In many research areas it is necessary to find differences between treatment groups with several variables. For example, studies of microarray data seek to find a significant difference in location parameters from zero or one for ratios thereof for each variable. However, in some studies a significant deviation of the difference in locations from zero (or 1 in terms of the ratio) is biologically meaningless. A relevant difference or ratio is sought in such cases.

**Results:**

This article addresses the use of relevance-shifted tests on ratios for a multivariate parallel two-sample group design. Two empirical procedures are proposed which embed the relevance-shifted test on ratios. As both procedures test a hypothesis for each variable, the resulting multiple testing problem has to be considered. Hence, the procedures include a multiplicity correction. Both procedures are extensions of available procedures for point null hypotheses achieving exact control of the familywise error rate. Whereas the shift of the null hypothesis alone would give straight-forward solutions, the problems that are the reason for the empirical considerations discussed here arise by the fact that the shift is considered in both directions and the whole parameter space in between these two limits has to be accepted as null hypothesis.

**Conclusion:**

The first algorithm to be discussed uses a permutation algorithm, and is appropriate for designs with a moderately large number of observations. However, many experiments have limited sample sizes. Then the second procedure might be more appropriate, where multiplicity is corrected according to a concept of data-driven order of hypotheses.

## Background

Nowadays, high-dimensional multivariate data are used in agriculture, biology and medicine. A recent example are microarray data, where two groups, for example normal and diseased tissue, are compared using tens of thousands of genes. The aim is to identify those genes with relevant over- or under-expression. Therefore, only two-sided tests are considered here. Nevertheless, directional one-sided relevance-shifted versions are also available [1]. Distinguishing between formal statistical significance and biological relevance is a frequently discussed issue [2]. One reason is that the commonly used point-zero null-hypothesis *H*_0 _: *μ*_2 _- *μ*_1 _= 0 is often biologically inappropriate, because depending on sample size and variance, biologically irrelevant small differences can be marked as statistically different. Therefore, the relevance-shifted null-hypothesis *H*_0 _: *μ*_2 _- *μ*_1 _- *δ *= 0 should be used. Hereby, the problem of the choice of *δ *appears [3]. Instead of absolute relevance margins, the use of relative margins may be more appropriate in some applications. For example, a compound will be declared potentially mutagenic in the Ames mutagenicity assay if the number of revertants is at least double of those in the control; this is the so-called two-fold rule [4]. Another example is the characterization of the anti-neoplastic activity of a new compound by its ratio of the mean tumor volume under treatment to that of the control [5]. For microarray experiments, a specific fold-change may be of interest as well. For example, Guo et al. [6] searched genes which were significant to the unadjusted *α *of 5% and have a fold change greater than 1.5. To analyze the relevance-shifted hypothesis in gene expression data, Li and Wong [7] propose using a confidence interval to estimate the fold change. However, this confidence interval requires the normality assumption and does not account for multiplicity. Both problems will be addressed in the subsequent proposals.

The problem occurs with the validation of normal assumptions for high dimensional data with small sample sizes. It seems to be hopeless to empirically characterize the distribution as multivariate normal. Hence nonparametric methods may be advantageous. In the current literature, examples for the analysis of multivariate studies using nonparametric methods can be found [8,9]. The present article focusses on nonparametric approaches as well. Relevance-shifted Wilcoxon rank statistics are used as basic test statistics in both approaches considered in this paper. Parametric test procedures for relevance-shifted hypotheses can be found in Frömke [1].

Since a local decision is provided for each of the thousands of genes, the resulting multiplicity problem has to be considered, too. Otherwise, the probability to falsely reject a null hypothesis increases dramatically. To overcome the problem of multiplicity, several approaches are discussed in the literature. Aside from the classical familywise error rate (FWER) – the probability to reject at least one true null hypothesis – the false discovery rate (FDR) introduced by Benjamini and Hochberg [10] is often used [11], giving the expected proportion of falsely rejected hypotheses among the set of all rejected hypotheses. According to the definitions, the FDR criterion usually delivers more 'significant' genes because – in contrast to the FWER – a small rate of falsely positive results is tolerated. Therefore, the main arguments in favour of the FDR (or against the FWER) are the low detection rate of the FWER procedures for high-dimensional data combined with a still sufficient type I error control for screening purposes. Nevertheless, many authors emphasize that the FWER criterion is necessary for confirmatory purposes [12-16]. Also the argument of the larger power of FDR procedures has to be qualified. As pointed out by Dudoit et al. [15] and Speed [16], the present FDR controlling procedures are usually based on independence assumptions between the single test statistics which are not acceptable in gene expression data (particularly, the Benjamini-Hochberg procedure) or they are corrected for that problem and are then computer intensive and/or so much reduced in their power that the advantage with respect to the FWER procedures is more or less lost. Here, we focus on two FWER controlling procedures (in the strong sense, i.e., keeping the FWER under any constellation of true and false local hypotheses) and we will demonstrate that they may be well applied also in high-dimensional data.

The simplest method to correct for multiplicity in both parametric and nonparametric settings is the *α*-adjustment of Bonferroni. Here the unadjusted *p*-values of the individual tests are compared with *α*/*m*, where *m *denotes the dimensionality of the data, that is, the number of observed variables. The modification by Holm [17] uses the threshold *α*/*m *only for the comparison with the smallest of the *m *individual *p*-values. The next smallest are compared to *α*/(*m *- 1), *α*/(*m *- 2), ..., *α*/1. If one of the *p*-values does not fall below the corresponding threshold, then the statistical procedure will stop and this null hypothesis as well as all succeeding ones will be accepted. However, even for low-dimensional data, the Bonferroni-type methods are known to be conservative. Furthermore, the potential improvement using the Holm method is minimal in high-dimensional data with only a small portion of variables with effects. One reason for the conservativeness is that the Bonferroni correction does not utilize the correlation structure of the variables. In rank tests with their discrete distributions, we have the additional problem that the procedures usually cannot fully exploit the prespecified error level but have to switch to the next possible *p*-value less than or equal to the given threshold. Particularly for very small sample sizes and a high dimension *m*, it might thus even be impossible to reject the null hypothesis using Bonferroni type methods. Both procedures extended in this paper utilize the covariance structure as they are based on permutations of the whole multivariate observation vectors although the technical procedure does not show that explicitly.

In the following text the two original procedures, which will be extended in this article, are briefly presented. The first procedure is the well-known permutation algorithm of Westfall and Young [18], as proposed by Dudoit et al. [19] for the analysis of microarray data. Just as the Bonferroni-Holm correction, this method is a step-down procedure. However, it consists of a permutation algorithm to compute the null distribution of the *p*-values. By permuting the variables, the algorithm takes their dependence structure into account. Given certain data conditions, above all not too small samples, this algorithm is probably the most powerful testing procedure for high-dimensional data in the current literature.

The second procedure is discussed in Kropf et al. [20]. This procedure belongs to the class of procedures with a data-driven order of hypotheses. These procedures consist of sequential testing of the variables at the unadjusted error level until the first nonsignificant result occurs. The order of testing is derived from the data themselves by means of selector statistics calculated for each variable (variables sorted for decreasing values of the selector statistics). The original procedure, which will be extended in this paper, is as follows:

1. For each of the *m *variables separately, compute the interquartile range using the combined data of the two samples. Order the *m *variables for decreasing values of their empirical interquartile ranges. The interquartile ranges serve as selector statistics.

2. Calculate the two-sample Wilcoxon test at the unadjusted level *α *in this order as long as significance is attained. Stop at the first nonsignificant result.

Assuming that all variables are measured on an equal scale and have similar variability within group, a large variability over the pooled samples for some of the variables could be a hint for large group differences. Therefore, the interquartile ranges of the pooled samples for each variable are used as selector statistics. The proof for the exact control of the familywise error rate utilizes, roughly speaking, the independence of rank and order statistics. If – contrary to the assumption – the variables have heterogeneous variability, then the procedure looses power. For example, Frömke [1] presents simulation studies, where the standard deviations vary by factor of 1.5 and the procedure looses approximately 10% power. The loss in power increases with increasing variability of standard deviations. However, the procedure controls the type I error in any case. A parametric counterpart of this procedure based on the theory of spherical distributions can be found in Kropf and Läuter [21].

Both of these nonparametric procedures have been shown to achieve the exact control of the familywise error rate under a point null hypothesis in the strong sense, where the observation vectors

(1) $$\begin{array}{c} x_{ik}={\left(x_{ik1},...,x_{ikj},...,x_{ikm}\right)}^{\prime }\sim F_{m}^{\left(i\right)}(x)\\ \left(i=1,2;k=1,...,n_{i}\right) \end{array}$$

belong to identical multivariate continuous distributions

(2) $$F_{m}^{\left(1\right)}(x)=F_{m}^{\left(2\right)}(x).$$

In this paper, we are interested in a slightly different situation. The model (1) is additionally restricted by the assumption that the independent and continuous vectors **x**_1*k *_and **x**_2*k *_only have positive components and that their distribution functions are equal except for a different scaling characterized by a vector ***θ ***= (*θ*_1_, ..., *θ*_*m*_)', that is

(3) $$F_{m}^{\left(1\right)}(x)=F_{m}^{\left(2\right)}(x/\theta ),$$

where the operator '/' indicates a componentwise division of vectors. Thus, *θ*_*j *_denotes the true ratio of the treatment medians of variable *j*.

For each of the *m *variables, it shall be tested, whether the two-sided null hypothesis

(4) *H*_0,*j *_: *θ*_*lower *_≤ *θ*_*j *_≤ *θ*_*upper*_

can be rejected in favor of the alternative

(5) *H*_1,*j *_: *θ*_*j *_<*θ*_*lower *_or *θ*_*j *_> *θ*_*upper*_,

where *θ*_*lower *_≤ 1 and *θ*_*upper *_≥ 1 denote the lower and the upper relevance threshold.

In both procedures considered here, this multiplicative model (3) is traced back to an additive one by a variablewise logarithmic transformation *y *= ln(*x*). So it changes to

(6) $$F_{m}^{*(1)}(y)=F_{m}^{*(2)}(y-\delta )$$

with

(7) ***δ ***= (*δ*_1_, ..., *δ*_*m*_)' and *δ*_*j *_= ln(*θ*_*j*_) (*j *= 1, ..., *m*)

and the null hypotheses are correspondingly transformed into

(8) $$H_{0,j}^{*}:\delta_{lower}\leq \delta_{j}\leq \delta_{upper}$$

and the alternative hypotheses are given as

(9) $$\begin{array}{ccc} H_{1,j}^{*}:\delta_{j}<\delta_{lower} & \text{or} & \delta_{j}>\delta_{upper} \end{array},$$

where *δ*_*lower *_= ln(*θ*_*lower*_) ≤ 0 and *δ*_*upper *_= ln(*θ*_*upper*_) ≥ 0 denote the lower and the upper relevance threshold. In practice, the choice of *δ*_*lower *_and *δ*_*upper *_is dependent on the experimental question. For example, in microarray experiments the thresholds can be set to -*δ*_*lower *_= *δ*_*upper *_= 0.4055, 0.6931 or 0.7885. This is equivalent to testing for a fold-change in gene expression of 1.5, 2 or 2.2 [6,22,23]. So an obvious approach would be to use the above mentioned two procedures after the logarithmic transformation and an additional shift by the relevance thresholds. However, this is associated with some problems. The shifted one-sided tests control the familywise error rate on the threshold which was used for shifting. But here we have two one-sided tests and two relevance thresholds, the lower and the upper one, and it is necessary to find some combination rule. It is likely that the second threshold (which is not used for the shift at that moment) is far enough from the first one so that a type I error caused by the one-sided test at the opposite border of the null space is unlikely. But the whole parameter space between both thresholds belongs to the null hypothesis as well and there is no proof that the two basic procedures control the type I error or are conservative under these conditions though one would expect a monotone behaviour of the rejection probability for increasing deviations from the exact null point. Finally, a correction is necessary for the selector statistic in the second procedure with data dependent sequential testing. Otherwise, variables with no shift or a only small one (but within the tolerance region) could have larger expected values for the selector than variables under the alternative hypothesis. The procedure would then loose its power by stopping prematurely.

The modifications of the exact procedures for point null hypotheses described in the following section have been adapted to these problems in an empirical manner. No exact proof exists for the control of the familywise error rate. Therefore, results of simulation experiments are presented after the detailed description of the modified procedures and their demonstration in examples. An R package for the methods is available [24].

## Results and Discussion

### Algorithm

#### Relevance-shifted permutation algorithm

We will first introduce a relevance-shifted modification of the permutation algorithm for step-down minP adjusted *p*-values of Westfall and Young [18] for point null hypotheses. More strictly speaking, we are starting from a proposal from Ge et al. [25] which delivers the same results as that of Westfall and Young but is less time consuming. Whereas the original algorithm requires two permutation runs, one for the calculation of raw *p*-values and a second one for multiplicity adjusting, Ge et al. [25] share the permutations of both runs.

In order to detect the deviation from the null hypothesis at both relevance thresholds, two passes are needed for each variable, one for relevant decrease and another one for relevant increase. Finally, out of the two one-sided *p*-values, a two-sided one is computed for each variable. The passes themselves consist of two parts. The relevance-shifted permuted unadjusted *p*-values from Wilcoxon's rank sum test are computed first. Then the unadjusted *p*-values are corrected for multiplicity. As mentioned above, we will use the log transformed observations and relevance thresholds.

The proposed algorithm is given here in detail for the *test on decrease*:

Part 1: Permutation algorithm for raw *p*-values

• Fix the thresholds *δ*_*lower *_= ln(*θ*_*lower*_) and *δ*_*upper *_= ln(*θ*_*upper*_).

• Create the pseudosample vectors $y_{ik}^{*}=\left(y_{ik1}^{*},...,y_{ikm}^{*}\right)$' with $y_{1kj}^{*}$ = *y*_1*kj *_+ *δ*_*lower *_and $y_{2kj}^{*}$ = *y*_2*kj *_(*i *= 1, 2; *k *= 1, ..., *n*_*i*_; *j *= 1, ..., *m*).

• In the *b*^*th *^permutation step, *b *= 0, ..., *B *(*b *= 0 corresponds to unpermuted data) do:

- For each variable, compute the one-sided Wilcoxon rank sum statistic *W*_1*b*_, ..., *W*_*mb *_for the pseudosamples:

$$W_{jb}=\sum_{k=1}^{n_{2}}r_{2jkb}$$

where ranks are computed over both groups and *r*_2*jkb *_denotes the *k*th ranked observation of the second group and the *j*th variable with the pseudosamples to test for decreases.

- Permute the *n*_1 _+ *n*_2 _= *N *pseudosample vectors $y_{ik}^{*}$ (*i *= 1, 2; *k *= 1, ..., *n*_*i*_).

• Calculate the one-sided raw *p*-values for hypothesis *H*_0,*j *_: *δ*_*j *_≥ *δ*_*lower *_as

(10) $$\begin{array}{c} p_{j,lower}^{*}=\frac{\#\{b:b>0\text{ and }W_{jb}\leq W_{j0}\}}{B}\\ \begin{array}{cc} \text{for} & j=1,...,m \end{array}. \end{array}$$

Part 2: Permutation algorithm for step-down minP adjusted *p*-values

• Re-number the *m *variables such that $p_{1,lower}^{*}\leq ...\leq p_{m,lower}^{*}.$

• Prepare three matrices for further computation:

The matrix **W **of size *m *× *B *includes the test statistics from the *B *permutation steps from Part 1 (renumbered and without the values for *b *= 0)

(11) $$W=\left(\begin{array}{ccccc} W_{11} & \cdots & W_{1b} & \cdots & W_{1B}\\ \vdots & & \vdots & & \vdots \\ W_{j1} & \cdots & W_{jb} & \cdots & W_{jB}\\ \vdots & & \vdots & & \vdots \\ W_{m1} & \cdots & W_{mb} & \cdots & W_{mB} \end{array}\right).$$

Two empty matrices **P **= (*p*_*jb*_) of size *m *× *B *and **Q **= (*q*_*jb*_) of size (*m *+ 1) × *B *are filled successively from the bottom to the top in the course of the following algorithm.

• Set *q*_*m*+1,*b *_= 1 for *b *= 1, ..., *B*.

• For *j *= *m*, *m *- 1, ..., 1 do:

Compute the *B *one-sided raw *p*-values *p*_*j*1_, ..., *p*_*jB *_for hypothesis *H*_0,*j *_(row *j *in matrix **P**) as

(12) $$p_{jb}=\frac{\#\{b^{\prime }:W_{jb^{\prime }}\leq W_{jb}\}}{B},$$

which is in row *j *of matrix **W **for each *W*_*jb *_the proportion of test statistics *W*_*jb' *_equal to or smaller than *W*_*jb*_.

• Determine the *j*th row of matrix **Q **as the successive minima

(13) *q*_*jb *_= min(*q*_*j*+1,*b*_, *p*_*jb*_), *b *= 1, ..., *B*.

Compute the adjusted *p*-value for hypothesis *H*_0,*j *_: *θ*_*j *_≥ *θ*_*lower*_:

(14) $${\tilde{p}}_{j,lower}^{*}=\frac{\#\{b:q_{jb}\leq p_{j,lower}^{*}\}}{B}.$$

• Enforce monotonicity of ${\tilde{p}}_{j,lower}^{*}$:

(15) $$\begin{array}{l} {\tilde{p}}_{1,lower}^{*}:={\tilde{p}}_{1,lower}^{*},\\ {\tilde{p}}_{j,lower}^{*}:=\max({\tilde{p}}_{j-1,lower}^{*},{\tilde{p}}_{j,lower}^{*})\\ \begin{array}{cc} \text{for} & j=2,...,m. \end{array} \end{array}$$

• Revoke the renumbering of the variables in the beginning of Part 2.

For the *test on increase*, repeat the entire procedure with the pseudosample vectors $y_{ik}^{*}=\left(y_{ik1}^{*},...,y_{ikm}^{*}\right)$', where $y_{1kj}^{*}$ = *y*_1*kj *_+ *δ*_*upper *_and $y_{2kj}^{*}$ = *y*_2*kj *_(*j *= 1, ..., *m*) and the rank sum test on increase to achieve the one-sided multiplicity adjusted *p*-values on increase ${\tilde{p}}_{j,upper}^{*}$. Finally, the two-sided adjusted *p*-values are given by ${\tilde{p}}_{j}^{*}=\min(2\cdot {\tilde{p}}_{j,lower}^{*},2\cdot {\tilde{p}}_{j,upper}^{*})$.

#### Procedure with a data-driven order of relevance-shifted hypotheses

An empirical extension for the nonparametric procedure of Kropf et al. [20] for relevance-shifted hypotheses will now be proposed:

• Select the two relevance thresholds *δ*_*lower *_= ln(*θ*_*lower*_) and *δ*_*upper *_= ln(*θ*_*upper*_).

• Determine the pseudosample vectors $y_{ik}^{*}=\left(y_{ik1}^{*},...,y_{ikm}^{*}\right)$ with $y_{1kj}^{*}$ = *y*_1*kj *_+ *δ*_*lower *_and $y_{2kj}^{*}$ = *y*_2*kj *_(*i *= 1, 2; *k *= 1, ..., *n*_*i*_; *j *= 1, ..., *m*) and calculate for each variable the one-sided Wilcoxon rank sum statistic *W*_*j*,*lower *_= $\sum_{k=1}^{n_{2}}r_{2jk}$ (*j *= 1, ..., *m*), again using the ranks determined over the two combined pseudosamples,

• Replace *δ*_*lower *_by *δ*_*upper *_and repeat exactly the former step to compute *W*_*j*,*upper *_(*j *= 1, ..., *m*).

• Use the permutation algorithm described in the previous procedure or suitable tables to derive the corresponding unadjusted one-sided *p*-values *p*_*j*,*lower *_and *p*_*j*,*upper*_, respectively, for the variablewise Wilcoxon statistics.

• Compute the unadjusted two-sided *p*-values *p*_*j *_as *p*_*j *_= min(2·*p*_*j*,*lower*_, 2·*p*_*j*,*upper*_) (*j *= 1, ..., *m*) for each variable.

• In order to prepare the determination of selector statistics, calculate the sample medians for the *j*th (logarithmic but not shifted) variable in sample 1 and 2, ${\tilde{y}}_{1j}$ and ${\tilde{y}}_{2j}$, respectively, and, once again, derive pseudosample values by

$$\begin{array}{l} y_{1kj}^{**}=\{\begin{array}{lll} y_{1kj}+\delta_{lower} & \text{if} & {\tilde{y}}_{2j}-{\tilde{y}}_{1j}<0\\ y_{1kj}+\delta_{upper} & \text{if} & {\tilde{y}}_{2j}-{\tilde{y}}_{1j}\geq 0 \end{array}\\ y_{2kj}^{**}=\{\begin{array}{lll} y_{2kj}-{\tilde{y}}_{2j}+{\tilde{y}}_{1j}+\delta_{upper} & \text{if} & 0\leq {\tilde{y}}_{2j}-{\tilde{y}}_{1j}<\delta_{upper}\\ y_{2kj}-{\tilde{y}}_{2j}+{\tilde{y}}_{1j}+\delta_{lower} & \text{if} & \delta_{lower}<{\tilde{y}}_{2j}-{\tilde{y}}_{1j}<0\\ y_{2kj} & \text{else}, & \end{array} \end{array}$$

(*k *= 1, ..., *n*_1 _or *k *= 1, ..., *n*_2_, respectively; *j *= 1, ..., *m*).

• Compute a selector statistic for each variable as the interquartile range *IRQ*_*j *_(difference of percentiles 75 and 25) from the combined sample values $y_{ikj}^{**}$, (*i *= 1, 2; *k *= 1, ..., *m*).

• Sort the *m p*-values *p*_*j *_for decreasing values of the corresponding selector statistics *IQR*_*j*_.

• In this order, compare the corresponding *p*-value with the unadjusted *α *for each variable *j*. The original variable has a significantly relevant ratio of medians if *p*_*j *_<*α *and all previously tested null hypotheses have been rejected, too.

• Stop at the first non-significance and accept the null hypothesis for all further variables.

The different formulae for the selector statistic depending on the difference of the two group medians (positive or negative, within or without the tolerance region) ensure an appropriate sorting of the variables giving the procedure a high power.

In the following sections, the Bonferroni-Holm procedure will be used for comparison. The unadjusted *p*-values will also be taken from two-sample Wilcoxon tests with the pseudosample values as in the above two methods. The one-sided *p*-values *p*_*j*,*lower *_and *p*_*j*,*upper *_will be determined separately for each of the *m *variables, each with the corresponding shift in the pseudosamples. These unadjusted *p*-values can be either taken from the first pass of the minP algorithm or from the second procedure. Then – as above – two-sided *p*-values will be calculated using *p*_*j *_= min(2·*p*_*j*,*lower*_, 2·*p*_*j*,*upper*_) (*j *= 1, ..., *m*) and will be used as the basis for the Bonferroni-Holm adjustment.

### Testing

#### Performance on simulated data

To confirm the control of the FWER, extended simulation studies were applied to the new procedures. A small part of the results for two-sided testing is shown in the following two tables. All scenarios were tested with three levels of relevance thresholds. For comparison with Kropf et al. [20], in one type of setting the thresholds were set to *θ*_*lower *_= *θ*_*upper *_= 1 (*δ*_*lower *_= *δ*_*upper *_= 0). In this case, the procedure with a data-driven order of relevance-shifted hypotheses reduces to the exact nonparametric procedure with a data-driven order of point-zero hypotheses for two unpaired samples applied to the logarithmized data. In the remaining two types of settings, the thresholds were set to $\theta_{lower}^{-1}$ = *θ*_*upper *_= 1.5

(*δ*_*lower *_= *δ*_*upper *_= 0.4055) or to the extreme case of $\theta_{lower}^{-1}$ = *θ*_*upper *_= 5 (*δ*_*lower *_= *δ*_*upper *_= 1.6094). Fifty observed variables were tested in all simulated situations.

To test if the procedures control the FWER in the weak sense, which is in the special case where all null hypotheses are true and the simulated FWER is less or equal to the selected *α*, 25 variables were generated with expected values of *μ*_1*j *_= *μ*_2*j*_/*θ*_*upper *_= 100 and true standard deviations of *σ*_1*j *_= *σ*_2*j*_/*θ*_*upper*_. The remaining 25 had *μ*_1*j*_/*θ*_*upper *_= *μ*_2*j *_= 100 and *σ*_1*j*_/*θ*_*upper *_= *σ*_2*j*_.

Furthermore, the control in the strong sense is important. In this case, the FWER is protected if some null hypotheses may be true and others false but the probability to reject any true null hypothesis is less or equal to *α*. For the assessment of the control, 45 variables were simulated under the null hypothesis and had the same true mean values as for the weak control; 22 were set to a non-relevant decrease and 23 to an increase. From the remaining five variables, two had a relevant ratio of treatment means with *μ*_1*j *_= 100·*θ*_*upper *_+ 50 and *μ*_2*j *_= 100 with *σ*_1*j *_= 10·*θ*_*upper *_+ 5, *σ*_2*j *_= 10 and the other three had *μ*_1*j *_= 100 and *μ*_2*j *_= 100·*θ*_*upper *_+ 50 with *σ*_1*j *_= 10, *σ*_2*j *_= 10·*θ*_*upper *_+ 5.

All variables had equal pairwise correlations *ρ *and equal variances 'on a logarithmic scale'. Together with the sample size per group, these parameters differed between the individual simulation settings and are noted in the table. If not stated otherwise, the random numbers were generated from a normal distribution, the nominal FWER was set *α *= 5% and the empirical FWER was computed with 10,000 simulation runs each. The modified Westfall-Young permutation algorithm is shown as 'permutation' in the following tables and figures and the procedure with a data-driven order of hypotheses is shown as 'selector'.

Table 1 presents the results of several simulation series for balanced multivariate normal samples at a nominal *α *level of 5% with varying relevance thresholds, sample sizes, variances, and pairwise correlation coefficients.

**Table 1:** Simulation results of the FWER for normal distributed data. This table shows simulation results of the relevance-shifted Westfall-Young permutation algorithm using 500,000 permutation runs ('permutation') and the procedure with a data-driven order of relevance-shifted hypotheses ('selector') for different levels of sample sizes, variances and correlation coefficients using normal distributed data.

				selector		permutation	
$\theta_{lower}^{-1}$ = *θ*_*upper*_	*n*_*i*_	*σ*_*ij*_	*ρ*	weak	strong	weak	strong
1	5	10	0.1	3.42	3.05	0	0
1.5	5	10	0.1	2.53	1.98	0	0
5	5	10	0.1	2.53	1.87	0	0
1	10	10	0.1	4.39	4.22	4.65	4.12
1.5	10	10	0.1	2.70	2.81	3.30	3.14
5	10	10	0.1	2.70	2.48	3.30	3.01
1	10	10	0.5	4.26	4.22	4.74	4.61
1.5	10	10	0.5	3.08	3.07	3.36	3.71
5	10	10	0.5	3.08	2.89	3.36	3.59
1	5	10	0.9	3.12	3.13	1.42	0.91
1.5	5	10	0.9	2.83	2.61	1.37	1.45
5	5	10	0.9	2.83	2.80	1.37	1.44
1	5	15	0.1	3.40	2.52	0	0
1.5	5	15	0.1	2.48	1.73	0	0
5	5	15	0.1	2.48	1.98	0	0
1	10	15	0.1	4.27	4.21	4.65	4.10
1.5	10	15	0.1	2.67	2.78	3.30	3.11
5	10	15	0.1	2.67	2.51	3.30	2.97
1	20	10	0.9	5.01	4.96	4.90	4.58
1.5	20	10	0.9	4.04	3.97	3.29	3.64
5	20	10	0.9	3.90	3.97	3.29	3.64

In Table 2, similar settings to the above were simulated. However, the random numbers were generated from a multivariate skewed distribution. For the generation of the random numbers, first univariate non-normal distributed samples with a priori selected expected value, standard deviation, skewness and kurtosis were created by application of a polynomial data transformation proposed by Fleishman [26]: A random variate *X *~ *N*(0, 1) is transformed into the polynomial *Y *= *a *+ *bX *+ *cX*^2 ^+ *dX*^3^. The dependence of the skewness $\gamma_{1}=\frac{E({\left(Y-\mu \right)}^{3})}{\sigma^{3}(Y)}$ and the kurtosis $\gamma_{2}=\frac{E({\left(Y-\mu \right)}^{4})}{\sigma^{4}(Y)}$ on the constants *a*, *b*, *c *and *d *is described in Fleishman's paper. An underlaying covariance matrix for the simulated vectors is created as follows: Let **x **denote an *m*-dimensional vector, where all components are iid with skewness *γ*_1 _and kurtosis *γ*_2_. Now determine a transformation matrix **R **of size *m *× *m*, such that **Σ **= **R'R **(for example with Cholesky decomposition). Then the transformed vector **y **= **R'x **has the variance-covariance matrix **Σ**. Using this method, sample vectors with *γ*_1 _= 2 and *γ*_2 _= 7 were produced for the simulation series in Table 2.

**Table 2:** Simulation results of the FWER for non-normal distributed data. This table shows the results for similar settings as in Table 2. Again, different levels of sample sizes, variances and correlation coefficients were tested, however non-normal distributed data was generated.

				selector		permutation	
$\theta_{lower}^{-1}$ = *θ*_*upper*_	*n*_*i*_	*σ*	*ρ*	weak	strong	weak	strong
1	5	10	0.1	3.31	2.87	0	0
1.5	5	10	0.1	2.40	1.57	0	0
5	5	10	0.1	2.40	1.78	0	0
1	10	10	0.1	4.15	4.32	4.88	4.18
1.5	10	10	0.1	2.48	2.64	3.46	3.42
5	10	10	0.1	2.48	2.37	3.46	3.15
1	5	10	0.9	3.19	2.96	0.80	0.39
1.5	5	10	0.9	2.78	2.45	0.83	1.12
5	5	10	0.9	2.78	2.64	0.84	1.07
1	10	10	0.9	4.33	4.48	4.65	4.25
1.5	10	10	0.9	3.51	3.59	3.26	0.33
5	10	10	0.9	3.51	3.49	3.26	0.33
1	5	5	0.9	3.16	3.13	0.80	0.46
1.5	5	5	0.9	2.77	2.8	0.84	1.12
5	5	5	0.9	2.77	2.44	0.84	1.12
1	5	15	0.9	3.17	2.73	0.80	0.31
1.5	5	15	0.9	2.76	2.44	0.80	1.09
5	5	15	0.9	2.76	2.56	0.84	1.03

The results in the tables show that the new procedures control the FWER empirically. Likewise, the FWER is protected for two-sided testing in further simulated situations, including other settings of the true mean values, skewed data, variances and correlations among the variables.

Extended results for one-sided testing using the procedure with a data-driven order of relevance-shifted hypotheses are also given [1]. Small increases of the FWER occurred in that case. The largest increase for the nominal *α *level of 5% was 6.3%. Error rates for the permutation algorithm corresponding to the one-sided case have not yet been been analyzed.

The control of the FWER is a premise of a statistical test. However, the aim of the experiments discussed here is to find variables which discriminate two kinds of treatments with a high probability. Hence, graphical representations of the simulation results in terms of the power of the new procedures compared to a standard technique will now be shown.

The simulation setting is nearly the same as for the control of the FWER in the strong sense. However, the setting of the expected values of variables under *H*_0 _was changed. For the control of the FWER, these variables had a ratio of means set to one of the margins of the null hypothesis because this choice resulted in the largest empirical FWER compared to variables with ratios closer to 1. A more realistic setting was selected for the simulation of the power, where a variable under *H*_0 _received a random ratio of means. This random value was a number *θ*_*lower *_≤ *τ *≤ *θ*_*upper*_. Two sets of values were created to generate *τ*. One set included all values between 1 and *θ*_*upper *_in steps of 0.05. To receive an equal amount of ratios in the second set, all values between 1 and $\theta_{lower}^{-1}$ in steps of 0.05 were computed and the second set took the inverse of these values. The sets were combined and *τ *was chosen separately for each variable. If *τ *< 1 then expectation values were set to *μ*_1*j *_= 100/*τ *and *μ*_2*j *_= 100 and the standard deviations were set to *σ*_1*j *_= *σ*_2*j*_/*τ*. Otherwise the true mean values were *μ*_1*j *_= 100 and *μ*_2*j *_= *τ*·100 with *σ*_1*j *_= *σ*_2*j*_/*τ*.

As for the simulations of the FWER, *α *= 5% and each result consisted of 10,000 simulation runs. In the tested scenarios, the thresholds were set to $\theta_{lower}^{-1}$ = *θ*_*upper *_= 2 (-*δ*_*lower *_= *δ*_*upper *_= 0.6931) and *σ*_*ij *_= 10.

All further settings of the parameters are given in the captions of the figures. The figures show the ratio of detected false null hypotheses as an estimation of the proportional power, which is defined as the average probability of rejecting the false null hypotheses [19]. The power of the exact relevance-shifted Wilcoxon rank sum test on ratio with the multiplicity correction of Bonferroni-Holm ('Bonferroni-Holm') is plotted together with simulation results of the two new procedures.

**Figure 1:**
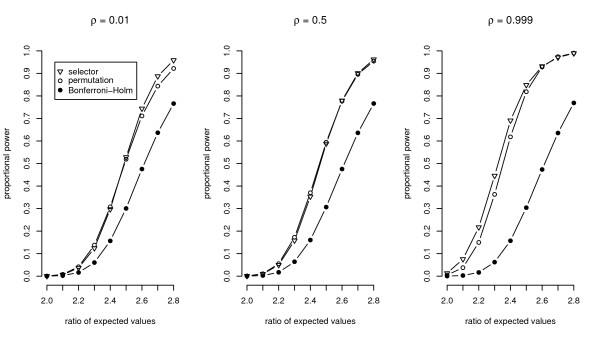
**Power for varying correlation structure among variables**. The three plots show the proportional power of the three procedures using a varying correlation structure among the variables. In each setting a sample size of *n*_*i *_= 7 was used.

**Figure 2:**
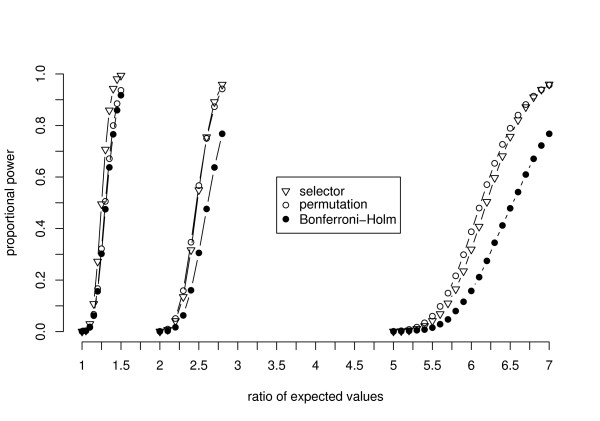
**Power for varying levels of relevance thresholds**. The figure shows the proportional power for varying levels of relevance thresholds. The power was computed using a sample size of *n*_*i *_= 7 and a correlation among the variables of *ρ *= 0.3. To simulate the power for the left curves the thresholds were set to *θ*_*lower *_= *θ*_*upper *_= 1, for the curves in the middle $\theta_{lower}^{-1}$ = *θ*_*upper *_= 2 were chosen and the power curves on the right were computed using $\theta_{lower}^{-1}$ = *θ*_*upper *_= 5.

It can be seen from Figure 1 that both new procedures achieve a higher power than the Bonferroni-Holm correction, irrespective of the correlation among the variables. While the power of the Bonferroni-Holm correction is constant for increasing correlation coefficients, the power of the new procedures increases. In Figure 2, the dependency of the three procedures on the relevance thresholds is shown. It can be clearly seen that the ratio of expected values has to be increased for all procedures in order to acquire a comparable power with increasing distance of the thresholds from the neutral value 1. In this and further simulation studies (results not shown here), the required ratio of expected values is approximately a multiple of the upper relevance threshold. The power is only smaller in the special case of $\theta_{lower}^{-1}$ = *θ*_*upper *_= 1, as here all ratios of variables under *H*_0 _are set to the margins of the thresholds. To achieve a power of around 50%, for example the procedure with a data-driven order of relevance-shifted hypotheses requires a ratio of expected values of 1.25 for $\theta_{lower}^{-1}$ = *θ*_*upper *_= 1. By multiplying this ratio of expected values with the upper relevance thresholds 2 or 5 (giving the ratios 2.5 and 6.25, respectively), the power is around 55% in both cases.

**Figure 3:**
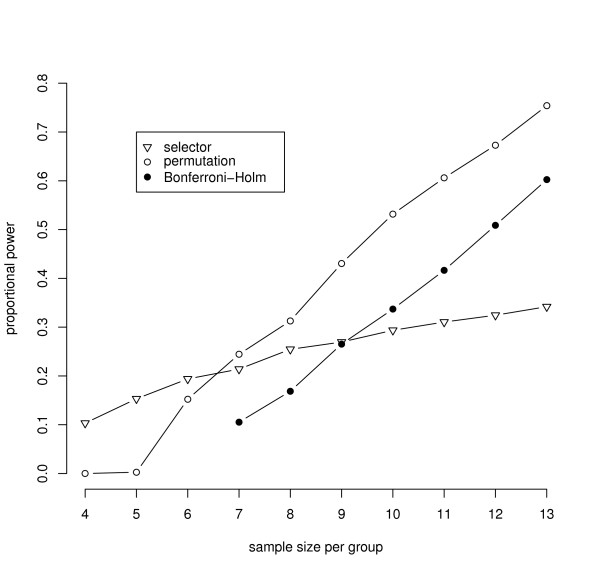
**Power for varying levels of sample sizes per group using 50 variables**. The figure shows the power for varying levels of samples sizes per group. All settings were simulated using a ratio of expected values for variables under *H*_1 _of 2.35 and a correlation among the variables of *ρ *= 0.3.

In Figure 3 the dependency of the three procedures on the sample sizes is shown. For small sample sizes, say 4 to 6, the procedure with a data-driven order of hypotheses is better than the permutation algorithm and the Bonferroni-Holm correction. With a sample size of 7 or more, the permutation algorithm achieves a higher proportional power. The Bonferroni-Holm correction can only be applied in this simulation setting if the sample size is at least 7. If the sample sizes are reduced to 6 per group, the smallest possible two-sided Bonferroni-Holm adjusted *p*-value is 0.108, and thus no significant variables can be achieved with *α *= 5%. The power of the Bonferroni-Holm correction also increases with increasing sample sizes. In the observed simulation setting a sample size of 10 is required to be better than the procedure with a data-driven order of hypotheses.

**Figure 4:**
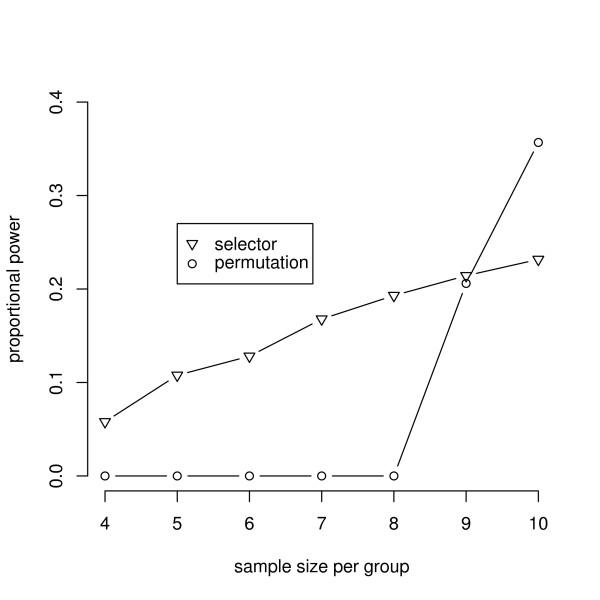
**Power for varying levels of sample sizes per group using 5,000 variables**. As in Figure 3 the power for varying levels of samples sizes per group are presented. The simulation settings were the same using in Figure 3. Only the expected values of the 50 variables under *H*_1 _were set to 2.5 and 1/2.5 and results consisted of 1,000 simulation runs. The simulations carried out for the permutation algorithm were time consuming. Therefore, the power computed for sample size 10 per group was simulated using 100 runs and the number of permutations in each run was restricted to 100,000.

In most microarray experiments several thousand variables are tested. Hence, simulations presented in Figure 4 were carried out including 5,000 variables as well. Basically, the simulation setting was the same as the setting presented in Figure 3. However, the number of variables was set to 5,000, where 50 variables were tested under *H*_1_. And as the power decreases with an increasing number of variables, the expected values were set to 1/2.5 and 2.5 for 25 variables under *H*_1 _each. The simulations of the permutation algorithm including 5,000 variables were time consuming. Therefore, simulations were carried out up to a sample size of 10 per group.

As in Figure 3, the procedure with a data-driven order of hypotheses is more powerful than the permutation algorithm if the sample sizes are small. However, using a larger sample size the permutation algorithm is preferable. The Bonferroni-Holm correction achieves no power, because the procedure is too discrete. If an experiment consists of 5,000 variables, a sample size of 12 per group is required to achieve a two-sided *p*-value of 3.7%. For example, using a sample size of 11 per group, the smallest achievable two-sided *p*-value is 14.2%. Irrespective of the effect size, this *p*-value cannot be less than *α *= 5%.

The choice of the procedure with the best power does not only depend on the sample size. In particular with an increasing *α*, the permutation algorithm and the Bonferroni-Holm correction are more powerful than the procedure with a data-driven order of hypotheses with sample sizes as low as 7 or 10. The choice is also dependent on the correlation among the variables as shown in Figure 1. Furthermore, the power of the procedure with a data-driven order of hypotheses reduces drastically in the case of variance heterogeneity among the variables. To be powerful, the procedure requires approximately homogeneous variances after the logarithmic transformation. Corresponding simulation results to these influences are given in Frömke [1]. Although the impact of a varying number of variables was not examined, it can be assumed to have significant effects as well.

### Implementation

#### Method comparison using a publicly available dataset

This section illustrates the application of the two procedures using a subset of the microarray study published by Khan et al. [27]. The entire data set consists of four subgroups of small, round blue cell tumors (SRBCTs) of childhood. Cell lines are available for all four subgroups, and biopsy material is available for two subgroups. The subset of the original study used here incorporates the biopsy material, which consists of 13 samples of the Ewing family of tumors (EWS) and 10 samples of the rhabdomyosarcoma (RMS). Furthermore, all 2,308 genes of the original data set will be analyzed. For the following analysis, significant two-fold under- or over expressions to an *α *= 5% are sought. Hence, the thresholds are set to $\theta_{lower}^{-1}$ = *θ*_*upper *_= 2 corresponding to -*δ*_*lower *_= *δ*_*upper *_= 0.6931.

The results of the relevance-shifted Westfall-Young permutation algorithm, the procedure with a data-driven order of relevance-shifted hypotheses and the Bonferroni-Holm correction are listed in Table 3. The last column provides a ranking number. These ranks are taken from the analysis methods supplement [27], where the top 96 genes were ranked according to importance using artificial neural network techniques.

**Table 3:** Results from the publicly available dataset. Results from the publicly available dataset: This table shows the results of the relevance-shifted Westfall-Young permutation algorithm using 500,000 permutation runs, the procedure with a data-driven order of relevance-shifted hypotheses and the Bonferroni-Holm correction. Note that the fourth and the sixth column are necessary for the the procedure with a data-driven order of relevance-shifted hypotheses only.

procedure	image id.	ratio	selector statistic	*p*-value	test decision	ranking
permutation	770394	0.051	-	0.00278	(reject *H*_0_)	6
	814260	0.032	-	0.00278	(reject *H*_0_)	75
	244618	24.918	-	0.00348	(reject *H*_0_)	7
	207274	4259.257	-	0.01983	(reject *H*_0_)	2
	43733	0.040	-	0.04832	(reject *H*_0_)	9
selector	207274	4259.257	6.881	0.00003	reject *H*_0_	2
	122159	169.102	6.204	0.02136	reject *H*_0_	40
	296448	1445.051	6.041	0.00145	reject *H*_0_	1
	34849	0.728	5.239	1.00000	accept *H*_0_, stop procedure	-
Bonferroni-Holm	770394	0.051	-	0.00403	(reject *H*_0_)	6
	244618	24.918	-	0.00403	(reject *H*_0_)	7
	814260	0.032	-	0.00403	(reject *H*_0_)	75

On top the table shows the results for the five significant genes found by the relevance-shifted Westfall-Young permutation algorithm after 500,000 permutation runs. In contrast, the procedure with a data-driven order of relevance-shifted hypotheses detects three significant genes, where one of them was also found using the above method. Three genes are also found with the Bonferroni-Holm adjustment. They are completely different genes compared to the former procedure, but they were also found using the modified Westfall-Young method.

In this analysis, the permutation algorithm detects more significant variables than both the procedure with a data-driven order of relevance-shifted hypotheses and the *α*-adjustment of Bonferroni-Holm. As shown in the former section, this can be explained with the general performance of these three methods for the present case of moderately large sample sizes in both groups. However, the procedure with a data-driven order of sequential testing is the only one that found the gene 296448, which according to Khan et al. [27] is the most important one.

## Conclusion

The comparison of two groups of individuals with many variables is a common problem in biological studies. In the current literature, procedures are proposed which perform local tests for each variable and correct for multiplicity. Most of these procedures test the point-zero or point-one null hypotheses of a difference or ratio in treatment effects of 0 or 1, respectively. A parametric procedure is available for relevance-shifted hypotheses [7]. In this article, two nonparametric procedures are proposed which perform a local relevance-shifted test on ratio on the two samples for each variable and include a multiplicity correction as well. They are extensions of the Westfall-Young permutation algorithm [18] and of a sequential procedure with data-driven order of hypotheses [20], which consider point-null hypotheses in their original form.

Both new procedures utilize the correlation structure. In the proofs of the original versions, this can be seen in the fact that they consider permutations of the whole observation vectors and not separate permutations for single variables. In the technical procedures, the influence of the correlation among the variables is not seen explicitly because univariate test statistics and selector functions are calculated. But it is present in the ordering of variables, which is part of both procedures in some way. When the variables are highly correlated, then the relation of their Wilcoxon test statistics or interquartile ranges effectively reflects the relation in the degree of violation of the corresponding null hypothesis. The less these correlations are, the more this relation is overlaid with random influences.

As not all modifications, applied to the point-null versions, could be covered by the theoretical considerations, simulation experiments were carried out for the control of the FWER and for the assessment of the power. In these experiments, the FWER was always controlled for the two-sided test versions discussed in this paper.

The power of the two new proposals and of the Bonferroni-Holm method was similar to the original procedures for point-null hypotheses (cf. Kropf et al. [20]). The procedure with data-driven sequential hypothesis testing uses a nonparametric measure of variability in the pooled samples as an additional source of information. This provides an advantage in small samples if the variances of the different variables are more or less homogeneous in the data. This advantage is lost and even reversed with increasing sample sizes. As discussed in Kropf et al. [20], this is due to the fact, that the probability to detect a difference in the unadjusted tests (which is the major input in the other test procedures) increases faster than the probability of the correct ordering of variables with and without deviations from the null hypothesis. Therefore, this ordering becomes the critical part in the sequential procedure for at least moderately large samples. However, data from microarray and proteomics experiments are commonly characterized by a very large number of variables and small sample sizes. The analysis of such experiments using standard multivariate approaches is inappropriate. The proposed procedures can be used instead, particularly if relevance shifted hypotheses are of interest.

## Authors' contributions

CF basically developed the modifications of the procedures, carried out the simulation studies and prepared the draft of the paper. LAH initiated the investigations, collected the relevant literature and essentially contributed to the modification of the permutation algorithm. SK delivered basic parts for the discussion of the multiple testing problem, contributed to the modification of the procedure with data-driven order and took part in the preparation of the final version of the paper. All authors read and approved the final manuscript.
